# The Relationship between *TP53* Gene Status and Carboxylesterase 2 Expression in Human Colorectal Cancer

**DOI:** 10.1155/2018/5280736

**Published:** 2018-01-31

**Authors:** Momoko Ishimine, Hyeon-Cheol Lee, Hirofumi Nakaoka, Hajime Orita, Toshiyuki Kobayashi, Konomi Mizuguchi, Mikumi Endo, Ituro Inoue, Koichi Sato, Takehiko Yokomizo

**Affiliations:** ^1^Department of Biochemistry, Juntendo University School of Medicine, Tokyo, Japan; ^2^Division of Human Genetics, National Institute of Genetics, Shizuoka, Japan; ^3^Department of Surgery, Juntendo Shizuoka Hospital, Juntendo University School of Medicine, Shizuoka, Japan; ^4^Shizuoka Medical Research Center for Disaster, Juntendo University, Shizuoka, Japan; ^5^Department of Pathology and Oncology, Juntendo University School of Medicine, Tokyo, Japan

## Abstract

Irinotecan (CPT-11) is an anticancer prodrug that is activated by the carboxylesterase CES2 and has been approved for the treatment of many types of solid tumors, including colorectal cancer. Recent studies with cell lines show that CES2 expression is regulated by the tumor suppressor protein p53. However, clinical evidence for this regulatory mechanism in cancer is lacking. In this study, we examined the relationship between *TP53* gene status and CES2 expression in human colorectal cancer. Most colorectal cancer specimens (70%; 26 of 37) showed lower CES2 mRNA levels (≥1.5-fold lower) than the adjacent normal tissue, and only 30% (12 of 37) showed similar (<1.5-fold lower) or higher CES2 mRNA levels. However, *TP53* gene sequencing revealed no relationship between CES2 downregulation and *TP53* mutational status. Moreover, while colorectal cancer cells expressing wild-type p53 exhibited p53-dependent upregulation of CES2, PRIMA-1^MET^, a drug that restores the transcriptional activity of mutant p53, failed to upregulate CES2 expression in cells with *TP53* missense mutations. These results, taken together, suggest that CES2 mRNA expression is decreased in human colorectal cancer independently of p53.

## 1. Introduction

Irinotecan (camptothecin-11 (CPT-11)) is a topoisomerase I inhibitor that has been used in the treatment of a wide spectrum of cancer, particularly colorectal cancer. Irinotecan is a prodrug, and its conversion to the active compound 7-ethyl-10-hydroxycamptothesin (SN-38) requires its hydrolysis by the carboxylesterase CES2. This enzyme exhibits a 60-fold higher hydrolytic efficiency against irinotecan than CES1, another human carboxylesterase isozyme [1], and the ability of colorectal tumors to hydrolyze irinotecan correlates with their expression of CES2, but not CES1 [2].

Although the liver is the major site for drug metabolism in general, it has been suggested that local (i.e., intratumoral) activation may also contribute to the efficacy of irinotecan in colorectal cancer [2–4]. Indeed, the colon may also serve as a major site for irinotecan activation since CES2 is highly expressed in this tissue, while the expression of the other carboxylesterase isozymes is relatively low [2]. A pharmacokinetic study revealed that the majority of intravenously injected irinotecan is excreted in the feces without being metabolized, indicating that a considerably high concentration of intact irinotecan is present in the colorectal lumen [5]. Thus, CES2 expression in colorectal cancer could be a key determinant of the therapeutic efficacy of this drug.

Recent studies suggest that CES2 expression is regulated by p53 in colorectal cancer cell lines [6, 7]. p53 is a tumor suppressor that is activated by a number of cellular stresses such as DNA damage, oxidative stress, and hypoxia. Approximately 50% of colorectal cancer bears missense mutations in *TP53*, the gene encoding p53. Immunohistochemistry (IHC) has been used in clinical settings to detect p53 mutations based on the premise that mutated p53 protein accumulates in the nucleus due to its impaired degradation [8]. Thus, if deregulated p53 signaling affects CES2 expression in colorectal cancer, accumulation of mutated p53 protein could be a potential diagnostic biomarker for predicting the therapeutic efficacy of irinotecan.

With these possibilities in mind, we investigated the relationship between *TP53* gene status and CES2 expression in colorectal cancer. Although CES2 expression was significantly lower in colorectal cancer specimens than in adjacent normal tissue, no clear correlation was observed between *TP53* gene status and CES2 expression. These results demonstrate the complexity of the regulatory mechanisms controlling CES2 expression in human colorectal cancer.

## 2. Materials and Methods

### 2.1. Cell Culture

The human colorectal cancer cell lines HCT116, HCT C, LoVo, RKO, and KM12C were provided by Dr. Tatsuro Irimura (Juntendo University). LS174T, Caco-2, and SW480 were from the American Type Culture Collection (ATCC). All cells were cultured at 37°C and 5% CO_2_ in a 1 : 1 (*v*/*v*) mixture of Dulbecco's modified Eagle's medium (Wako) and Ham's F-12 medium (Wako) supplemented with 10% fetal bovine serum and penicillin/streptomycin. Cells expressing wild-type p53 (HCT116, HCT C, LoVo, RKO, and LS174T) [9, 10] and p53-null Caco-2 [11] cells were seeded at 2 × 10^5^ cells/well in 6-well plates and incubated for 24 hours. The cells were then treated with 10 *μ*M nutlin-3a (AdooQ BioScience) for 24 hours. Cells expressing mutant p53 (KM12C and SW480) [12, 13] were seeded at 4 × 10^5^ cells/well in 6-well plates and incubated for 24 hours. The cells were then treated with 120 *μ*M PRIMA-1^MET^ (Tocris Bioscience) for 24 hours. The cells were washed twice with PBS and harvested by scraping.

### 2.2. Tumor and Adjacent Normal Tissue Specimens

All tumors and adjacent normal tissues were obtained from patients undergoing surgery at the Department of Surgery, Juntendo Shizuoka Hospital, Juntendo University School of Medicine. The study was approved by the Medical Research Ethics Committee of Juntendo Shizuoka Hospital (ethics approval number 457), and written informed consent was obtained from all patients.

### 2.3. Real-Time Reverse Transcriptase PCR

Total RNA from cell lines and tissues was extracted using the RNeasy Mini Kit (Qiagen) according to the manufacturer's protocol. One microgram (cells) or 0.2 *μ*g (tissue) of total RNA was reverse transcribed with random hexamers using SuperScript III (ThermoFisher Scientific) to generate cDNA. Semiquantitative real-time PCR was performed using FastStart Essential DNA Green Master (Roche) on a LightCycler 96 (Roche). *GAPDH* (cell lines except for LoVo) and *18S rRNA* (LoVo and tissues) were used as reference genes. PCR reactions were performed in duplicate for all genes. The relative expression of each gene was calculated using the 2^−ΔΔCt^ method. The sequences of the primers used were as follows: *CES2* forward 5′-GTAGCACATTTTCAGTGTTCC-3′ and reverse 5′-GTAGTTGCCCCCAAAGAA-3′, *p21* forward 5′-GATTTCTACCACTCCAAACGCC-3′ and reverse 5′-AGAAGATGTAGAGCGGGC-3′, *Noxa* forward 5′-GCTGGAAGTCGAGTGTGCTA-3′ and reverse 5′-CCTGAGCAGAAGAGTTTGGA-3′, *GAPDH* forward 5′-TGCCCTCAACGACCACTTTG-3′ and reverse 5′-CTCTTCCTCTTGTGCTCTTGCTG-3′, and *18S rRNA* forward 5′-GTAACCCGTTGAACCCCATT-3′ and reverse 5′-CCATCCAATCGGTAGTAGCG-3′.

### 2.4. Sequencing

DNA was extracted from each tumor sample using the AllPrep DNA/RNA Mini Kit (Qiagen) according to the manufacturer's protocol. Sanger sequencing was performed using the BigDye Terminator Cycle Sequencing V3.1 Ready Reaction kit (ThermoFisher Scientific) on an ABI 3130xl Genetic Analyzer (Applied Biosystems). The oligonucleotide primers used are shown in Table S1. *TP53* genotypes of the colorectal cancer were categorized as nonfunctional, partially functional, or functional using the International Agency of Research on Cancer (IARC) *TP53* mutation database (release R18) [14].

### 2.5. Plasmid Construction and Transfection

Human CES2 cDNA with a stop codon was amplified by PCR from NCI-H226 cell cDNA library with primers hCES2_cloning_forward 5′-TAGTTAAGCTTATGACTGCTCAGTCCCGCTC-3′ and hCES2_cloning_reverse 5′-GGCCCTCTAGACTACAGCTCTGTGTGTCTCT-3′ and was cloned into the pcDNA3.1/Myc-His vector using HindIII and XbaI sites. HEK293T cells were transfected with the plasmid DNA or empty vector control using polyethylenimine Max (MW 40,000) (Polysciences, Warrington, PA).

### 2.6. Gel-Based Activity-Based Protein Profiling Analysis

Cells and tissues were homogenized in PBS and centrifuged at 100,000 ×g for 45 min at 4°C. The soluble proteomes (15 *μ*g in 30 *μ*L of PBS) were incubated with 1 *μ*M fluorophosphonate-rhodamine probe for 30 min at 37°C. After 30 min, reactions were quenched with 4x SDS/PAGE loading buffer (reducing), separated by SDS/PAGE [10% (*w*/*v*) acrylamide], and visualized using a Typhoon FLA 9500 (GE Healthcare Life Sciences). Images were analyzed with ImageQuant TL software (GE Healthcare Life Sciences). Rhodamine fluorescence is shown in gray scale.

## 3. Results and Discussion

### 3.1. Upregulation of CES2 in p53 Wild-Type Colorectal Cancer Cells

**Figure 1:**
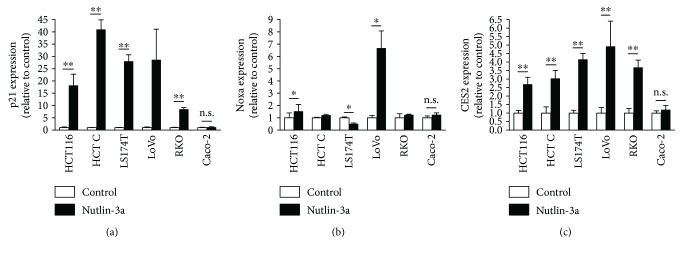
The expression of p21 (a), Noxa (b), and CES2 (c) was quantified by real-time reverse transcriptase PCR. Human colorectal cancer cell lines with wild-type p53 (HCT116, HCT C, LS174T, LoVo, and RKO) and p53-null Caco-2 cells were treated with 10 *μ*M nutlin-3a for 24 hours. For LoVo cells, *18S rRNA* was used as the reference gene. For other cell types, *GAPDH* was used as the reference gene. Data represent the mean values ± SEM (*n* = 3–4). ^∗^ *P* < 0.05; ^∗∗^ *P* < 0.01; n.s.: no significance. A paired two-tailed *t*-test was used.

We first asked whether p53 activation enhances CES2 expression. Several colorectal cancer cell lines expressing wild-type p53 (HCT116, HCT C, LS174T, LoVo, and RKO) [9, 10] and a p53-null cell line (Caco-2) [11] were treated with nutlin-3a, which inhibits the interaction between p53 and the E3 ubiquitin ligase MDM2 and thereby directly activates p53 signaling without genotoxic side effects [15]. The expression of p21, a downstream target of p53, increased following nutlin-3a treatment in all p53 wild-type cell lines tested, indicating enhanced p53 pathway activation in those cells (Figure 1(a)) [16]. Nutlin-3a also enhanced the expression of Noxa, another downstream target of p53, in HCT116 and LoVo cells (Figure 1(b)) [16]. Furthermore, nutlin-3a significantly increased the expression of CES2 in all of the cell lines expressing wild-type p53 (Figure 1(c)). However, the expression of these genes did not change in response to nutlin-3a in p53-null Caco-2 cells (Figures 1(a)–1(c)). These results provide further evidence that CES2 expression is positively regulated by the p53 pathway in colorectal cancer cells.

### 3.2. CES2 Expression and Activity in Human Colorectal Cancer

Approximately 50% of colorectal cancer is reported to have missense mutations in the *TP53* gene. Most of those mutations are found in the region encoding the DNA-binding domain. Such mutated p53 not only has reduced DNA-binding capacity but also exerts a dominant negative effect on wild-type p53 [17], thereby severely compromising p53 function. The deregulation of the p53 pathway caused by these missense mutations affects the expression of numerous downstream target genes in cancer [16, 18, 19].

**Figure 2:**
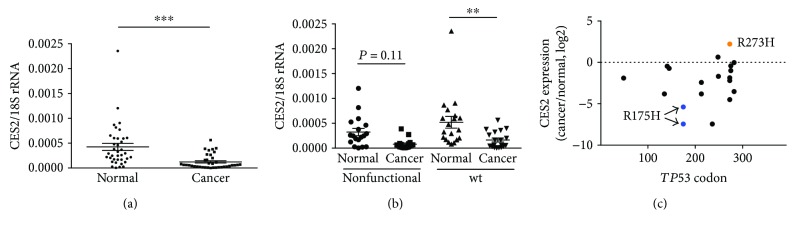
CES2 expression in human colorectal cancer and adjacent normal tissues was quantified by real-time reverse transcriptase PCR. (a) CES2 mRNA was significantly decreased in the tumor tissue. *18S rRNA* was used as a reference gene. ^∗∗∗^ *P* < 0.001 by paired two-tailed *t*-test. (b) The CES2 expression levels were compared between tumors with *TP53* mutations generating nonfunctional p53 protein and tumors without *TP53* mutations. ^∗∗^ *P* < 0.01. A Tukey-Kramer test was used. (c) The relationship between CES2 expression and the position of *TP53* mutations. The plot indicates the codon distribution of the *TP53* missense mutations (*x*-axis), and the samples' corresponding CES2 expression levels (*y*-axis). The blue dots and the arrows indicate the samples with the R175H mutation. The orange dot indicates the sample with the R273H mutation.

To evaluate the relationship between CES2 expression and *TP53* gene status in colorectal cancer, CES2 mRNA levels were compared in colorectal cancer specimens and adjacent normal tissue collected from 37 patients. Most of the tumor samples (70%; 26 of 37) had lower CES2 expression than adjacent normal tissue (≥1.5-fold decrease), and only 30% (12 of 37) had similar (<1.5-fold decrease) or higher CES2 mRNA levels (Table S2, Figure 2(a)). This result is largely in agreement with that of a previous study that reported that colorectal cancer expressed lower levels of CES2 protein than adjacent normal tissue [3].

Next, the *TP53* gene in each of the tumor samples was sequenced using an ABI 3130xl Genetic Analyzer. *TP53* mutations that reportedly result in a severe loss of p53 function were identified in 51% of the cancers (19 of 37) (Table S2), and all except one (18 of 19) were located in the region that encodes the DNA-binding domain of the p53 protein. Unexpectedly, the CES2 mRNA levels in the tumors with *TP53* mutations resulting in nonfunctional p53 protein were comparable to those in the tumors without *TP53* mutations (*P* = 0.85) (Figure 2(b)). These data suggest that the reduced expression of CES2 in colorectal cancer occurs independently of *TP53* gene status. Indeed, while the two tumors with the R175H mutation (patients 5 and 6), one of the most common pathogenic loss-of-function *TP53* mutations in colorectal cancer, showed a dramatic reduction in CES2 mRNA (42-fold and 174-fold, resp.) (Figure 2(c)), a sample with another common pathogenic mutation, R273H (patient number 14), exhibited higher CES2 expression (5-fold) in the tumor than in the adjacent normal tissue (Figure 2(c)). This also suggests the existence of alternative mechanisms enhancing CES2 expression even when the p53 pathway is inactivated.

**Figure 3:**
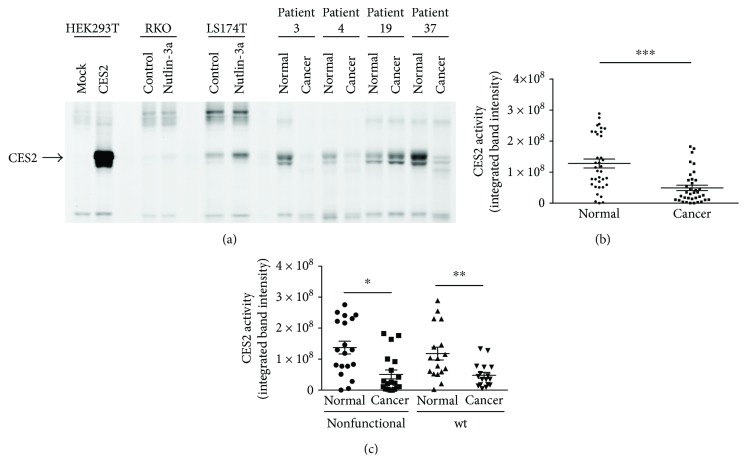
CES2 activity in human colorectal cancer and adjacent normal tissues was quantified by ABPP. (a) CES2 activities of HEK293T cells (mock versus CES2 overexpression), colorectal cancer cells (RKO and LS174T cells; control versus nutlin-3a), and representative human colorectal samples were shown. (b) CES2 activity was significantly decreased in the tumor tissue. ^∗∗∗^ *P* < 0.001 by paired two-tailed *t*-test. (c) The CES2 activities were compared between tumors with *TP53* mutations generating nonfunctional p53 protein and tumors without *TP53* mutations. ^∗^ *P* < 0.05; ^∗∗^ *P* < 0.01. A Tukey-Kramer test was used.

We also measured the CES2 activity of the colorectal samples by activity-based protein profiling (ABPP) using a serine hydrolase activity probe fluorophosphonate-rhodamine [20]. HEK293T cells expressing CES2 and colorectal cancer cells treated with nutlin-3a exhibited increased CES2 activity (Figure 3(a)). Similar to its expression, CES2 activity was lower in the tumors than in the adjacent normal tissue (Figures 3(a) and 3(b)), and the reduction of CES2 activity also occurred independently of *TP53* gene status (*P* = 0.9995) (Figure 3(c)).

We finally examined the relationship between CES2 expression and p21 expression. We found that p21 expression was significantly reduced in the tumors compared with adjacent normal tissue (Figure S1 (a)) and that the reduction was also independent of the presence of *TP53* mutation (*P* = 0.9997) (Figure S1 (b)). Notably, there was a significant positive correlation between p21 and CES2 expression even in the tumors with nonfunctional p53 (Table S3). It has been shown that p21 expression can be induced by various stress signals in a p53-independent fashion [21]. These data, taken together, suggest that CES2 expression is regulated in a p53-independent manner that also controls p21 expression in colorectal cancer.

### 3.3. CES2 Expression in Colorectal Cancer Cells with TP53 Missense Mutations

**Figure 4:**
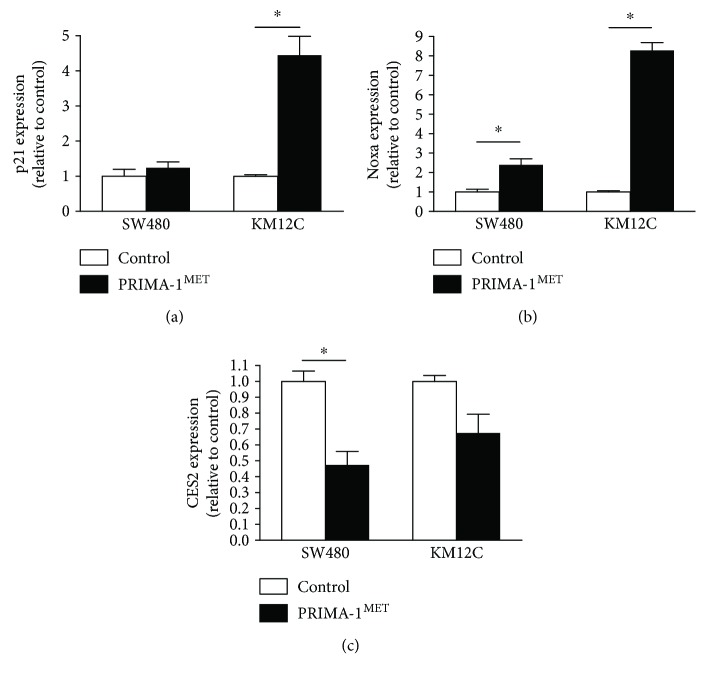
The expression of p21 (a), Noxa (b), and CES2 (c) was quantified by real-time reverse transcriptase PCR. Human colorectal cancer cell lines with *TP53* missense mutations (SW480 and KM12C) were treated with 120 *μ*M PRIMA-1^MET^ for 24 hours. *GAPDH* was used as a reference gene. Data represent the mean values ± SEM (*n* = 3–4). ^∗^ *P* < 0.05; n.s.: no significance. A paired two-tailed *t*-test was used.

Recent studies identified small molecules that reactivate mutant p53 [22–25]. Although there was no clear relationship between CES2 expression and the presence of *TP53* missense mutations in the colorectal cancer specimens in this study, restoration of p53 function might reactivate the p53 pathway, resulting in an upregulation of CES2 expression. To test this hypothesis, colorectal cancer cells harboring mutant p53 were treated with PRIMA-1^MET^, a p53-reactivating drug. This agent has been tested in a phase I/IIa clinical trial for the treatment of hematologic malignancies and prostate cancer [26] and is currently being tested in a phase Ib/II clinical trial in patients with high-grade serous ovarian carcinoma. Among the cell lines tested, p21 expression was upregulated following PRIMA-1^MET^ treatment in KM12C (p53 H179R) [12] cells (Figure 4(a)), and Noxa expression was upregulated in response to PRIMA-1^MET^ in both SW480 (p53 R273H/P309S) [13] and KM12C cells (Figure 4(b)), suggesting that the reactivation of the p53 pathway occurred in these cells. However, CES2 expression was decreased in these cells after p53 pathway reactivation (Figure 4(c)). These data, taken together, provide further evidence that the regulation of CES2 expression is not straightforward and that mechanism(s) other than p53 are likely to be involved.

Shang et al. previously showed that CES2 expression is regulated by the xenobiotic-sensing transcription factor Pregnane X receptor (PXR) in HepG2 human hepatoma cells [27]. However, PXR agonism did not lead to upregulation of CES2 expression in colorectal cancer cells (LoVo, SW480, and KM12C) (data not shown), suggesting distinct mechanism(s) of CES2 regulation in colorectal cancer cells. Recent studies show that CES2 activates not only irinotecan but also other anticancer drugs such as gemcitabine [28], capecitabine [29], and pentyl PABC-Doxaz (PPD) [30]. Thus, understanding how CES2 expression is regulated in normal and cancerous cells will expand the utility of these anticancer drugs.

## 4. Conclusions

Most of the colorectal cancer specimens analyzed in this study showed a considerable reduction in CES2 expression compared with adjacent normal tissue. However, there was no obvious correlation between *TP53* gene status and the reduction in CES2 expression. Thus, given the role of local CES2 expression in the activation of irinotecan, direct measurement of CES2 expression and/or activity (rather than *TP53* mutational status or p53 protein expression) may be an effective method to predict the efficacy of irinotecan in colorectal cancer. Our results, however, do not rule out a potential role for wild-type p53, which could upregulate CES2 when activated by anticancer drugs. Further studies should be performed to fully understand the regulatory mechanisms of CES2 expression in cancer and the contribution of p53.

## Supplementary Materials

Supplementary Materials Table S1: PCR primers used for *TP53* sequencing. Table S2: colorectal cancer specimens analyzed in this study. Figure S1: p21 expression in human colorectal cancer and adjacent normal tissues was quantified by real-time reverse transcriptase PCR. (a) p21 mRNA was significantly decreased in the tumor tissue. 18S rRNA was used as a reference gene. ^∗∗∗^ *P* < 0.001 by paired two-tailed *t*-test. (b) The p21 expression levels were compared between tumors with *TP53* mutations generating nonfunctional p53 protein and tumors without *TP53* mutations. ^∗∗∗^ *P* < 0.001. A Tukey-Kramer test was used. Table S3: correlation between p21 and CES2 expression.
