# Supplementary material for: The Relationship between TP53 Gene Status and Carboxylesterase 2 Expression in Human Colorectal Cancer
Source: Dis Markers. 2018 Jan 31;2018:5280736. doi: 10.1155/2018/5280736 (PMC5831679; doi:10.1155/2018/5280736)
Supplement: Supplementary Materials — Table S1: PCR primers used for TP53 sequencing. Table S2: colorectal cancer specimens analyzed in this study. Figure S1: p21 expression in human colorectal cancer and adjacent normal tissues was quantified by real-time reverse transcriptase PCR. (a) p21 mRNA was significantly decreased in the tumor tissue. 18S rRNA was used as a reference gene. ∗∗∗ P < 0.001 by paired two-tailed t-test. (b) The p21 expression levels were compared between tumors with TP53 mutations generating nonfunctional p53 protein and tumors without TP53 mutations. ∗∗∗ P < 0.001. A Tukey-Kramer test was used. Table S3: correlation between p21 and CES2 expression. [file 5280736.f1.pdf]

Table S1. PCR primers used for *TP53* sequencing.

**Target**	**Forward**	**Reverse**	**Reference**
Exon 1	CACAGCTCTGGCTTGCAGA	AGCGATTTTCCCGAGCTGA	Liu & Bodmer[1]
Exon 2	AGCTGTCTCAGACACTGGCA	GAGCAGAAAGTCAGTCCCATG	Liu & Bodmer[1]
Exons 3-4	AGACCTATGGAAACTGTGAGTGGA	GAAGCCTAAGGGTGAAGAGGA	Liu & Bodmer[1]
Exons 5-6	CGCTAGTGGGTTGCAGGA	CACTGACAACCACCCTTAAC	Liu & Bodmer[1]
Exon 7	CTGCTTGCCACAGGTCTC	TGGATGGGTAGTAGTATGGAAG	Liu & Bodmer[1]
Exons 8-9	GTTGGGAGTAGATGGAGCCT	GGCATTTTGAGTGTTAGACTG	Liu & Bodmer[1]
Exon 10	GGTACTTGAAGTGCAGTTTCT	CAGCTGCCTTTGACCATGAA	Verselis *et al.*[2]
Exon 11	TCCCGTTGTCCCAGCCTT	TAACCCTTAACTGCAAGAACAT	Liu & Bodmer[1]

1. Liu Y, Bodmer WF. Analysis of P53 mutations and their expression in 56 colorectal cancer cell lines. Proc Natl Acad Sci U S A. 103: 976-981 (2006).
2. Verselis SJ, Rheinwald JG, Fraumeni JF Jr, Li FP. Novel p53 splice site mutations in three families with Li-Fraumeni syndrome. Oncogene. 19: 4230-4235 (2000).

Table S2: Colorectal cancer specimens analysed in this study

Patient No.	Age	Sex	Lesion Site	Histologic Type	Stage	T	N	M	Preoperative Chemotherapy	CES2/18S rRNA(ratio to Normal tissue)	TP53 genotype	TP53 Functionality
1	78	F	sigmoid colon	tub2>tub1	2	3	0	0		0.305660069	P72R,H179Y	partially functional(H179Y)
2	69	M	rectum	tub1,2>pap>>muc	3a	3	1	0		0.035158078		
3	76	F	ascending colon	pap/tub	1	2	0	0		0.050765775	P72R	
4	55	M	rectum	tub2>muc	2	3	0	0		0.138696184	P72R	
5	76	F	rectum	tub1>tub2	2	3	0	0		0.024013675	P72R,R175H	non-functional(R175H)
6	77	M	sigmoid colon	tub2>tub1	3a	3	1	0		0.005758864	P72R,R175H	non-functional(R175H)
7	66	F	ascending colon	tub2>muc	2	3	0	0		0.102237757	P72R	
8	63	M	rectum	tub2>tub1>por	2	3	0	0		0.726986259	P72R,C141Y	non-functional(C141Y)
9	66	M	sigmoid colon	tub2>tub1>por	2	3	0	0		0.607097442	P72R,L145P	non-functional(L145P)
10	65	M	ascending colon	tub2	2	3	0	0		0.246558176	P72R	
11	78	M	transverse colon	tub2>tub1	3a	3	1	0		0.986232704	R282W	non-functional(R282W)
12	64	F	rectum	tub2>tub1>por	2	4	0	0		0.071297732	P72R,C135F	non-functional(C135F)
13	84	F	sigmoid colon	tub1>tub2	1	2	0	0		0.079109787	P72R	
14	64	M	rectum	tub2>tub1>muc	4	2	0	1(left lung)		4.658934346	R273H	non-functional(R273H)
15	53	M	ascending colon	tub2>muc	2	3	0	0		0.267943366	D49H,P72R	non-functional(D49H)
16	68	F	transverse colon	tub2>muc	4	3	1	1 (p)		0.277392368	P72R,R273C	non-functional(R273C)
17	60	M	rectum	tub2>por	3a	4b	1	0		0.087171479	P72R,R282W	non-functional(R282W)
18	67	F	sigmoid colon	tub2>tub1>por	3a	3	1	0		0.243163737	P72R	
19	67	M	sigmoid colon	por>tub2	3b	4b	2	0		2.173469725	P72R,T211I	partially functional(T211I)
20	76	M	sigmoid colon, metastatic liver cancer	tub2>muc	4	3	0	1a(H1)		0.901250463	P72R	
21	42	M	rectum(Ra)	por>tub2>muc	3b	3	1	0		1.094293701	P72R	
22	45	F	rectum	por>tub, pap	3a	3	1	0	FOLFOX	0.258816231	P72R	
23	71	F	rectum	tub2	2	3	0	0		0.312082637	P72R,R249W	non-functional(R249W)
24	68	M	rectum	tub2	2	3	0	0		0.063372467	P72R	
25	51	F	rectum	tub1>tub2	2	3	0	0		0.503477775	P72R,C275F	non-functional(C275F)
26	65	F	rectum	tub2>tub1>por	4	3	1	0		1.569168196	E11Q,P72R	
27	62	M	rectum(Rs)	tub2	2	2	0	0	FOLFOX+BV	0.005758864	P72R,Y236C	non-functional(Y236C)
28	75	M	sigmoid colon	tub2	4	3	3	1a(P)		0.21915143	P72R,R273C	non-functional(R273C)
29	40	M	rectum	tub2	3a	2	1	0		0.048697786	P72R	
30	61	F	sigmoid colon, rectum	tub2	3a	3	1	0		0.752623374		
31	81	M	rectum	tub2>tub1	2	3	0	0		0.128514228	P72R	
32	80	F	transverse colon	tub2,por	3a	1b	1	0		0.071793647	P72R,R213Q	non-functional(R213Q)
33	65	F	transverse colon	tub2	4	3	1	1b(P,OTH)		0.186856156	P72R,R213Q	non-functional(R213Q)
34	66	F	ascending colon	tub2	2	3	0	0		1.569168196	R156C,Q192K,R248W	partially functional(R156C), functional(Q192K), non-functional(R248W)
35	58	M	ascending colon	tub2>tub1>por	4	3	2	1b(H3,PUL,LYM)		0.747424624	P72R,C275Y	non-functional(C275Y)
36	70	M	ascending colon	tub2>tub1	1	2	0	0		4.316912946	P72R	
37	75	M	transverse colon	tub2>tub1>por	1	2	0	0		0.044194174	P72R,R273C	non-functional(R273C)

Histologic Type tub: tublar adenocarcinoma(well differentiated type:tub1, moderately differentiated type:tub2) pap: papillary adenocarcinoma muc: mucinous adenocarcinoma por: poorly differentiated adenocarcinoma(solid type:por1, non-solid type:por2)

T: depth of tumor invasion N: lymph node metastasis M: distant metastasis

M: distant metastasis   P:peritoneum H:liver OTH:other PUL:lung LYM:lymph node

Preopetative Chemotherapy FOLFOX:Infusional 5-FU+Leucovorin+Oxaliplatin BV:Bevacizmab

Figure S1

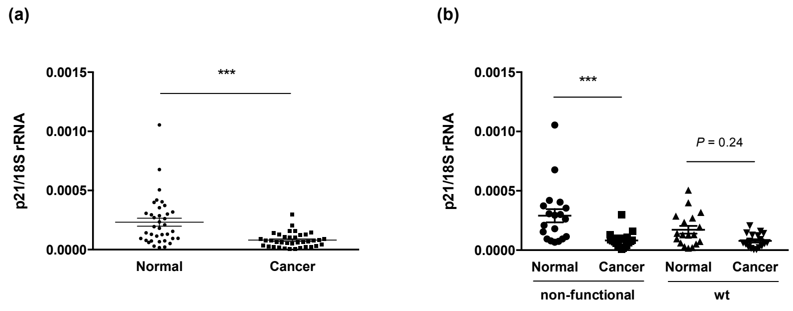

Figure S1. p21 expression in human colorectal cancers and adjacent normal tissues was quantified by real-time reverse transcriptase PCR. (a) p21 mRNA was significantly decreased in the tumor tissue. 18S rRNA was used as a reference gene. ***, $P < 0.001$ by paired, two-tailed t-test. (b) The p21 expression levels were compared between tumors with *TP53* mutations generating non-functional p53 protein and tumors without *TP53* mutations. ***, $P < 0.001$. A Tukey-Kramer test was used.

Table S3. Correlation between p21 and CES2 expression

TP53 gene status	Tissue	Pearson's *r*	95% CI	*P* value
non-Functional	Normal tissue	0.8901849	0.7319082 – 0.9573205	3.319E-07
	Cancer	0.7019436	0.3636890 – 0.8766509	0.000808
wt	Normal tissue	0.7337255	0.4058974 – 0.8942621	0.0005287
	Cancer	0.2390073	-0.2564825 – 0.6350186	0.2390073
